# CRISPR/Cas9-mediated mutagenesis of *BnaAOG1s* reveals functional divergence in silique and seed development in *Brassica napus* L

**DOI:** 10.3389/fpls.2026.1862587

**Published:** 2026-07-20

**Authors:** Jiaxu Xiao, Xiaonan Guo, Aoli Liao, Zhiru Bao, Jianwei Gu

**Affiliations:** Hubei Key Laboratory of Resource Utilization and Quality Control of Characteristic Crops, College of Life Science and Technology, Hubei Engineering University, Xiaogan, Hubei, China

**Keywords:** *Brassica napus*, CRISPR/Cas9, functional divergence, seed development, silique development

## Abstract

Oilseed rape (*Brassica napus* L.) is a major oil crop, and both silique and seed size are critical determinants of yield. In *Arabidopsis thaliana*, mutation of the *ABORTED GAMETOPHYTE 1* (*AOG1*) gene leads to severe defects in gametophyte development and a pronounced reduction in silique length. However, the function of *AOG1* homologs in rapeseed remains uncharacterized. In this study, four homologous copies of *AOG1* were identified in *B. napus*: *BnaAOG1.A03*, *BnaAOG1.A10*, *BnaAOG1.C03*, and *BnaAOG1.C09*. Then a systematic analysis that considered the physiochemical properties, evolution, conserved motifs, gene structure, and cis-regulatory elements of *BnaAOG1s* family members was conducted. Utilizing the CRISPR/Cas9 system, two of these copies, *BnaAOG1.A03* and *BnaAOG1.C03*, were targeted, and homozygous double mutants were generated. Unlike the Arabidopsis ortholog, the *BnaAOG1s* were specifically expressed during seed development. Phenotypic evaluation revealed that the double mutants did not exhibit significant changes in silique length, seed number per silique, or thousand-seed weight compared to the wild type. These results indicate that *BnaAOG1.A03* and *BnaAOG1.C03* are not individually essential for silique and seed development in *B. napus*. Their functions may be compensated by other homologous copies or have undergone divergence during polyploidization. This investigation provides a valuable case for functional analysis of homologous genes in polyploid crops.

## Introduction

*Brassica napus* L. (AACC, 2n = 38) is one of the most important oil crops worldwide. It originated from interspecific hybridization between *Brassica rapa* (AA, 2n = 20) and *Brassica oleracea* (CC, 2n = 18), followed by spontaneous chromosome doubling (Zhao et al., 2021). In China, the planting area of rapeseed accounts for approximately one third of the global total, and its annual oil production contributes to over 50% of domestic edible vegetable oil. Consequently, improvements in rapeseed yield are directly pertinent to national food and oil security (Qu et al., 2025). The yield of rapeseed is determined by three key components: thousand-seed weight (TSW), seed number per silique (SPS), and effective silique number per plant. The silique, as the organ that bears seeds, not only provides physical protection but also serves as a major photosynthetic organ during late seed development, supplying nutrients for seed maturation. Therefore, silique-related traits (including length, width, thickness, and volume) are closely associated with seed size and weight and have long been important targets for genetic improvement of yield in rapeseed (Hua et al., 2011; Yang et al., 2018; Zhou et al., 2021; Zhao et al., 2021).

Over the past two decades, quantitative trait locus (QTL) mapping for yield−related traits has been extensively conducted in *B. napus* using double haploid (DH) and recombinant inbred line (RIL) populations. Zhao et al. (2021) performed QTL analysis of five silique−related traits in a RIL population of 189 lines across seven environments, identifying 120 consensus QTLs covering all chromosomes except C5. Among them, 13 QTLs were stably detected in multiple environments, explaining 4.38% to 13.0% of the phenotypic variation. Fu et al. (2015) also identified multiple QTLs for silique length and seed weight through comparative QTL analysis. Several genes underlying these QTLs have been functionally characterized. *BnaA9.ARF18*, the first identified negative regulator of silique length and seed weight in *B. napus*, was cloned via map−based cloning and association analysis; it controls silique length and seed size by repressing auxin−responsive genes (Liu et al., 2015). *BnaA9.CYP78A9*, encoding a P450 monooxygenase, promotes cell elongation in silique valves and increases silique length and seed weight when highly expressed (Shi et al., 2019). In addition, *BnaC9.SMG7b*, a positive regulator of SPS, was found to be deleted in the low−SPS parent HZ396 (Li et al., 2015). The causal gene of the major QTL *cqSL−C7* was identified as *BnaC7.ROT3*, which regulates silique length by affecting cell elongation in the silique epidermis (Zhou et al., 2022). Despite these advances, the number of functionally characterized genes controlling silique and seed size in rapeseed remains limited, and the underlying molecular regulatory networks are still poorly understood.

In the model plant *Arabidopsis thaliana*, studies on silique development and gametophyte formation provide important references for rapeseed research. Through screening of a Ds insertion mutant collection, Cui et al. (2015) identified a previously uncharacterized gene, *AOG1* (*At5g57790*). Mutation of *AOG1* caused severe defects in both male and female gametophyte development: male gametophytes arrested at the uninucleate microspore stage with about 60% of pollen grains degenerating; female gametophytes arrested at the uninucleate embryo sac stage; and silique length was markedly shortened, with seed set reduced to only 8.77% (compared to 99.24% in the wild type). *AOG1* encodes a 216−amino acid nuclear−localized protein with no significant sequence similarity to any documented proteins, and is highly expressed in reproductive tissues, especially in developing pollen grains and ovules (Cui et al., 2015). The essential role of AOG1 in Arabidopsis gametophyte development suggests that its homologous genes in rapeseed might also participate in regulating silique and seed development. However, *B. napus* is an allotetraploid with a complex genome containing extensive gene redundancy, chromosomal duplications, and rearrangements (Song et al., 2020). In polyploids, homoeologs may exhibit functional redundancy, subfunctionalization, or neofunctionalization (Adams and Wendel, 2005; Hu et al., 2026; Ryan and Adams, 2026; Lynch and Conery, 2000; Flagel and Wendel, 2009). The functional analysis of duplicated genes in polyploids is challenged by allelic dosage uncertainty and inaccurate genotyping, making it difficult to predict the functions of rapeseed homoeologs solely based on knowledge from model plants. Therefore, direct functional studies of *AOG1* homoeologs in *B. napus* are necessary to understand their evolutionary fate and regulatory roles in this polyploid crop.

In this study, four homoeologous copies of *AOG1* were identified in the *B. napus* genome and their sequence features and conserved domains were systematically characterized. The CRISPR/Cas9 system was employed to target two copies, *BnaAOG1.A03* and *BnaAOG1.C03*, which are highly expressed during seed development, and homozygous double mutants were obtained. Through detailed evaluation of silique length, SPS, and TSW, a preliminary assessment of the functions of these genes in silique and seed development in rapeseed is provided herein.

## Materials and methods

### Plant materials and growth conditions

The rapeseed (*B. napus*) inbred line “Xiaoyun” (Y127) served as the recipient material for genetic transformation. This line is a facultative spring type with a short growth cycle under a comprehensive speed breeding system (approximately 57 days per generation) and shows no adverse phenotypes, making it suitable for functional gene studies (Song et al., 2022). Seeds of wild−type Xiaoyun were surface−sterilized with 75% ethanol for 1 min, followed by 50% commercial bleach (1:1 dilution with sterile water) for 6 min, rinsed 3–5 times with sterile water, and sown on germination medium (M0: MS medium + 30 g·L^-1^ sucrose + 8 g·L^-1^ agar, pH 5.84–5.88). They were incubated at 22 °C in the dark for 6 days before transformation.

Tissue culture was performed in a growth chamber at 22 °C under a 16 h light/8 h dark photoperiod with a light intensity of 50–100 μmol·m^-2^·s^-1^. Greenhouse conditions were maintained at 22 °C, 22 h light/2 h dark, 60–70% relative humidity, and an LED light source (red:green:blue = 6:2:2) with an intensity of about 950 μmol·m^-2^·s^-1^ to enable rapid generation advancement (Song et al., 2022).

### Homolog identification and sequence analysis

Homologous copies of AtAOG1 in the Xiaoyun genome were identified by BLASTP searches against the BnGDXY database (http://yanglab.hzau.edu.cn/BnGDXY/#/blast) using the AtAOG1 protein sequence as query. Homologous copies of AtAOG1 in the *Brassica juncea*, *Brassica carinata*, *Brassica nigra*, *Brassica rapa*, and *Brassica oleracea* genomes were identified by BLASTP searches against the BnIR database (http://yanglab.hzau.edu.cn/BnTIR/). The filter parameters were set as E value ≦ 1.0e-5, Query coverage ≧ 50% and Identity ≧ 40%. Other AOG1 homologous protein sequences were identified by BLASTP searches against the UniProt database (https://www.uniprot.org/). Candidate sequences were verified for conserved domains using NCBI CDD (https://www.ncbi.nlm.nih.gov/cdd). Molecular weight (MW) and theoretical isoelectric point (pI), instability index, aliphatic index, and grand average of hydropathicity (GRAVY) were calculated with ExPASy ProtParam (https://web.expasy.org/protparam/). The location information of proteins was predicted by Cell-PLoc 2.0 (http://www.csbio.sjtu.edu.cn/bioinf/Cell-PLoc-2/). Multiple sequence alignments were performed using Geneious software with default parameters.

### *Cis-regulatory* element analysis

The 2000 bp upstream sequences of the translation start site (ATG) of five AOG1 family genes were retrieved from genomic databases: *AtAOG1* from Arabidopsis thaliana (TAIR), and four *B. napus* homologs from BnGDXY. The *cis-regulatory* elements were predicted by PlantCARE (http://bioinformatics.psb.ugent.be/webtools/plantcare/html/) with manual curation. A total of 15 cis-elements belonging to 8 functional categories were systematically scanned (**Supplementary Table 1**). Pattern matching was performed using regular expressions with case-insensitive search. Overlapping matches were counted independently.

### Phylogenetic analysis

The 18 protein sequences of AOG1 gene family members among 9 plants were first aligned using the ClustalW algorithm with default parameters (gap opening penalty = 10; gap extension penalty = 0.2 in multiple alignments; delay divergent cutoff = 30%). We constructed a phylogenetic tree based on the ML (Maximum Likelihood) method with a bootstrap of 1000 replicates and visualized using the iTOL tool (https://itol.embl.de/).

### Gene structure and conserved motif analysis

Genomic DNA and coding sequence (CDS) of each gene were retrieved from BnGDXY and NCBI. Exon–intron structures were visualized using GSDS2.0 (http://gsds.cbi.pku.edu.cn/) based on alignment of CDS with genomic sequences. Conserved motifs were identified with MEME (http://meme-suite.org/tools/meme) using 20 motifs and a width of 6–50 amino acids. Motif annotation was performed with Tomtom (https://meme-suite.org/meme/tools/tomtom) (Li et al., 2021).

### Expression profiling

Expression levels (FPKM) of BnaAOG1s in various tissues (root, stem, leaf, flower bud, flower, silique wall, and seed) of the *B. napus* cultivar Zhongshuang 11 (ZS11) were obtained from the BnIR database (http://yanglab.hzau.edu.cn/BnTIR/). Raw transcriptome data were extracted, and expression profiles were generated.

### Target site design and vector construction

A single guide RNA (sgRNA) targeting the conserved coding regions of *BnaAOG1.A03* and *BnaAOG1.C03* was designed using CRISPR−P 2.0 (http://crispr.hzau.edu.cn/CRISPR2/) (Liu et al., 2017). The target sequence was 5′−GGAGGCATCTGTATCTGCGG−3′ (located in the first exon, PAM motif CGG). The sgRNA oligonucleotide and its complement (with added overhangs complementary to BsaI sticky ends) were annealed to form a dimer. The dimer was ligated into the BsaI−linearized pRGEB32−BnU6C01 vector via Golden Gate assembly using T4 ligase. The ligation product was transformed into Escherichia coli DH5α competent cells, and positive clones were selected on kanamycin (50 mg·L^-1^) and confirmed by sequencing. The verified plasmid was electroporated into Agrobacterium tumefaciens strain GV3101 (1, 800 V), and positive agrobacterial clones were selected and stored at –80 °C.

### Agrobacterium−mediated genetic transformation

Hypocotyl transformation of rapeseed was performed following a previously described protocol (Dai et al., 2020). Briefly, sterilized seeds were germinated on M0 medium at 24 °C in the dark for 6 days. Hypocotyls of 6−day−old seedlings were excised into 0.8–1.0 cm explants and infected with the Agrobacterium suspension (OD_600_ = 0.4–0.6) for 15 min, followed by co−cultivation for 36–48 h. Explants were then transferred to selection medium (M2) for 20 days, followed by subculture on differentiation medium (M3) every 15–20 days until green shoots appeared. Shoots with intact meristems were transferred to rooting medium (M4) for root induction. Well−rooted plantlets were transferred to the greenhouse.

### Identification of transgenic plants and analysis of gene editing

Genomic DNA from the transgenic plants was extracted using the CTAB method and stored in a 96-well plate for subsequent positive identification and genetic typing (Doyle and Doyle, 1987). The PCR amplification system was prepared using 2×Taq Master Mix, and primers BnU6C01-Cas9-F1/R1 and BnU6C01-Cas9-F2/R2 were used to detect the presence of transgene in the plants (**Supplementary Table 2**). Specific primers were designed for *BnaAOG1.A03* and *BnaAOG1.C03* and adapter sequences were appended to both ends of the forward and reverse primers, serving as the first-round PCR primers (Liu et al., 2019, sgRNA-4285-F/R, **Supplementary Table 2**). A set of Hi-TOM primers with index sequences was designed for the second-round PCR (GTseq_i5/i7, designed by Wuhan GenoSeq Technology Co., Ltd.; **Supplementary Table 2**). Using the genomic DNA of positive transgenic plants as a template, the first-round PCR amplification was performed, followed by using the resulting products as templates for the second-round PCR amplification. The second-round PCR products were then sequenced using the Illumina HiSeq platform, and the sequencing data was analyzed to determine the sgRNA integration status in various transgenic plant samples. The entire sequencing and data analysis process was conducted by Wuhan GenoSeq Technology Co., Ltd.

### Agronomic trait evaluation

Homozygous T_1_ mutant lines and wild−type Xiaoyun were grown in the greenhouse under identical conditions. Each group contained 10–15 plants for final phenotypic statistical analysis. Agronomic traits evaluated included plant height, main inflorescence length, silique length, effective silique number per plant, seed number per silique, and thousand−seed weight. Silique length was measured with a ruler on 10 siliques of similar size from the main stem per plant, and the average was calculated. Seed number per silique was counted after manual threshing of 10 siliques per plant. Thousand−seed weight was determined by collecting all seeds of each plant and removing impurities and then weighing the seeds on a SC-G seed detector (Hangzhou Wanshen Detection Technology Co., Ltd., Hangzhou, China).

### Statistical analysis

All data are presented as mean ± standard deviation (SD) from at least three biological replicates. Student’s *T*−test was used to assess significance, with *p* < 0.05 considered significant and *p* < 0.01 highly significant. Statistical analyses and graph generation were performed using Microsoft Excel 2019.

## Results

### Identification and molecular characterization of *BnaAOG1s*

Four homoeologous copies of *AOG1* were identified in the Xiaoyun genome by BLASTP (E−value ≤ 1e−10) using the AtAOG1 protein sequence. Based on their chromosomal positions, they were named *BnaAOG1.A03* (*BnaA03G0116700XY*), *BnaAOG1.A10* (*BnaA10G0136800XY*), *BnaAOG1.C03* (*BnaC03G0127800XY*), and *BnaAOG1.C09* (*BnaC09G0426400XY*).

**Figure 1:**
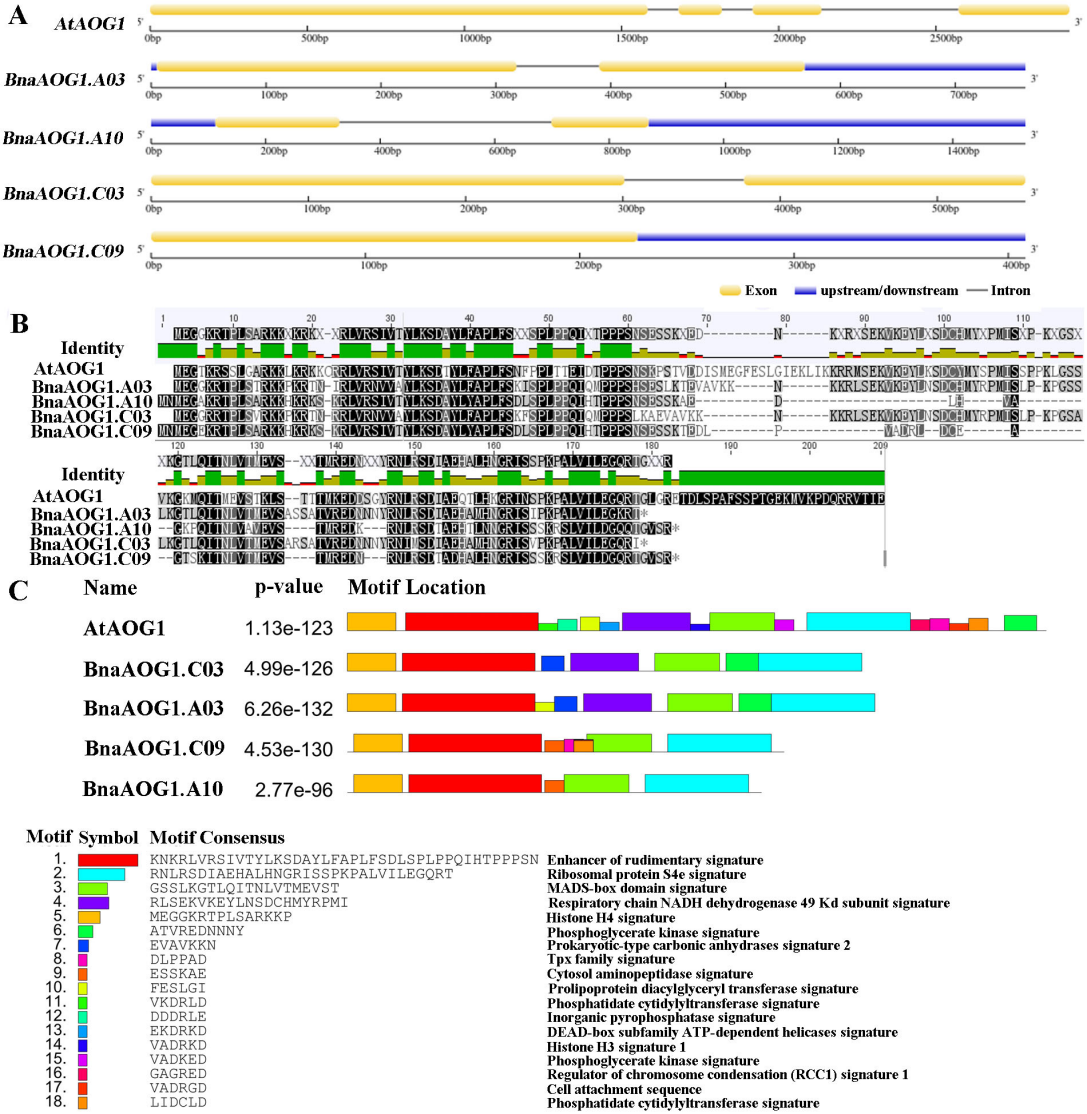
Molecular characterization of *AtAOG1* homologous in *B. napus*. **(A)**, The exon-intron organizations of AtAOG1 and homologous in *B. napus*. **(B)**, The sequence alignment of AtAOG1 and homologous in *B. napus*. **(C)**, the motif analysis of AtAOG1 and homologous in *B. napus*.

Genomic structure analysis revealed significant differences between the homologous genes in rapeseed and AtAOG1 (**Figure 1A**). The coding sequence lengths of *BnaAOG1.A03*, *BnaAOG1.A10*, *BnaAOG1.C03*, and *BnaAOG1.C09* are 492 bp, 387 bp, 480 bp, and 408 bp, encoding 163, 128, 159, and 138 amino acids, respectively (**Table 1**). The calculated molecular weights are 18.32 kDa, 14.23 kDa, 17.98 kDa, and 15.10 kDa, and the theoretical pI values are 10.23, 10.23, 10.44, and 9.80, respectively. All BnaAOG1 proteins are basic proteins, similar to AtAOG1 (pI 10.47) (**Table 1**). All proteins are hydrophilic (GRAVY < 0) and the instability index (II) indicate these are relatively unstable proteins *in vitro* (**Table 1**). Protein sequence alignment revealed that BnaAOG1.A03 and BnaAOG1.C03 share 56.3% and 54.5% similarity with AtAOG1, whereas BnaAOG1.A10 and BnaAOG1.C09 exhibit lower similarity (45.5% and 46.6%) (**Table 1**; **Figure 1B**). Further comparison demonstrates that BnaAOG1.A03 and BnaAOG1.C03 are the most similar among the four copies (85.3%), and the identity value of BnaAOG1.A10 and BnaAOG1.C09 is 82.4%, while the A10 and C09 copies only show about 50–60% similarity to the A03/C03 copies (**Supplementary Figure 1**). Conserved domain analysis indicated that neither AtAOG1 nor any of the BnaAOG1 proteins contains known conserved functional domains, consistent with the report by Cui et al. (2015).

**Table 1:** Comparison of the physicochemical properties of BnaAOG1s and AtAOG1.

Gene name	CDS (bp)	Protein (aa)	MW	pI	GRAVY	II	AI	Similarity(%)	Location
AtAOG1	651	216	24.46	10.47	-0.586	57.52	21.8	100	Nucl
BnaAOG1.A03	492	163	18.32	10.23	-0.507	52.42	39.1	56.3	Nucl
BnaAOG1.A10	387	128	14.23	10.23	-0.591	59.49	41	45.5	Nucl
BnaAOG1.C03	480	159	17.98	10.44	-0.781	55.08	48.4	54.5	Nucl
BnaAOG1.C09	408	138	15.1	9.8	-0.729	56.99	34	46.6	Nucl

MW, Molecular weight (kDa); pI, isoelectric point; II, Instability Index; AI, Aliphatic Index; nucl, Nucleus.

### Phylogenetic analysis, conserved motifs, and gene structures of BnaAOG1 family members

**Figure 2:**
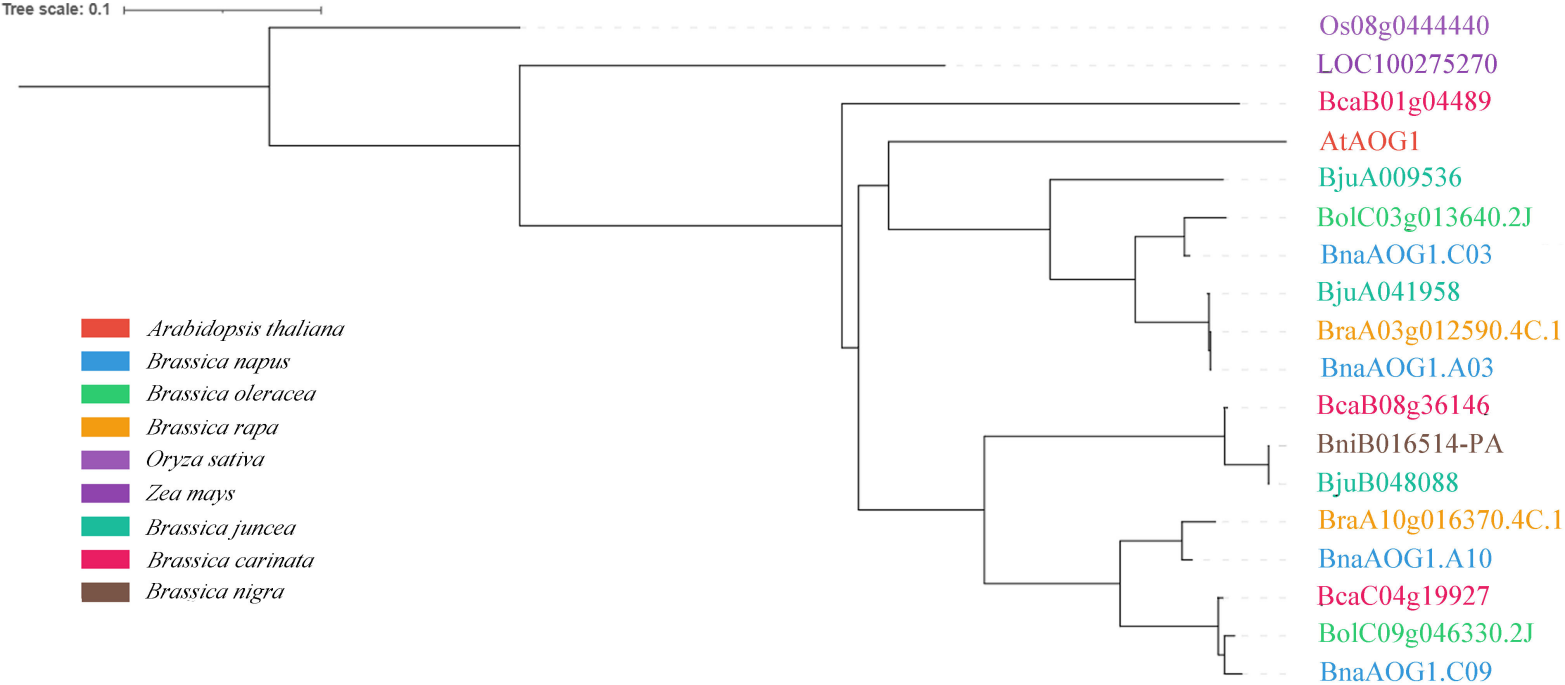
Phylogenetic tree of plant AOG1s protein sequences. The bootstrap number was 1000. Different colored fonts represent different species.

To understand the phylogenetic relationships of plant AOG1s, we used four BnaAOG1s, two BolAOG1s, two BraAOG1s, three BjuAOG1s, three BcaAOG1s, one BniAOG1s, one AtAOG1, one OsAOG1 and one ZmAOG1 to construct a phylogenetic tree. The tree reveals distinct evolutionary relationships among the 18 AOG1 homologs, with clear separation between monocot and dicot lineages (**Figure 2**). Furthermore, BnaAOG1.A03/C03 and BnaAOG1.A10/C09 form two distinct evolutionary branches.

Motif analysis using MEME identified 18 motifs (motifs 1-18) in AtAOG1 and the BnaAOG1s. AtAOG1 contained 16 motifs, whereas the BnaAOG1s had markedly fewer motifs (**Figure 1C**). Specifically, BnaAOG1.A03 and BnaAOG1.C03 contained 8 and 7 motifs, respectively, while BnaAOG1.A10 and BnaAOG1.C09 retained 5 and 7 motifs, with differences in motif composition among copies (**Figure 1C**). These results suggest functional divergence of BnaAOG1s during evolution.

### *Cis-regulatory* element analysis

**Figure 3:**
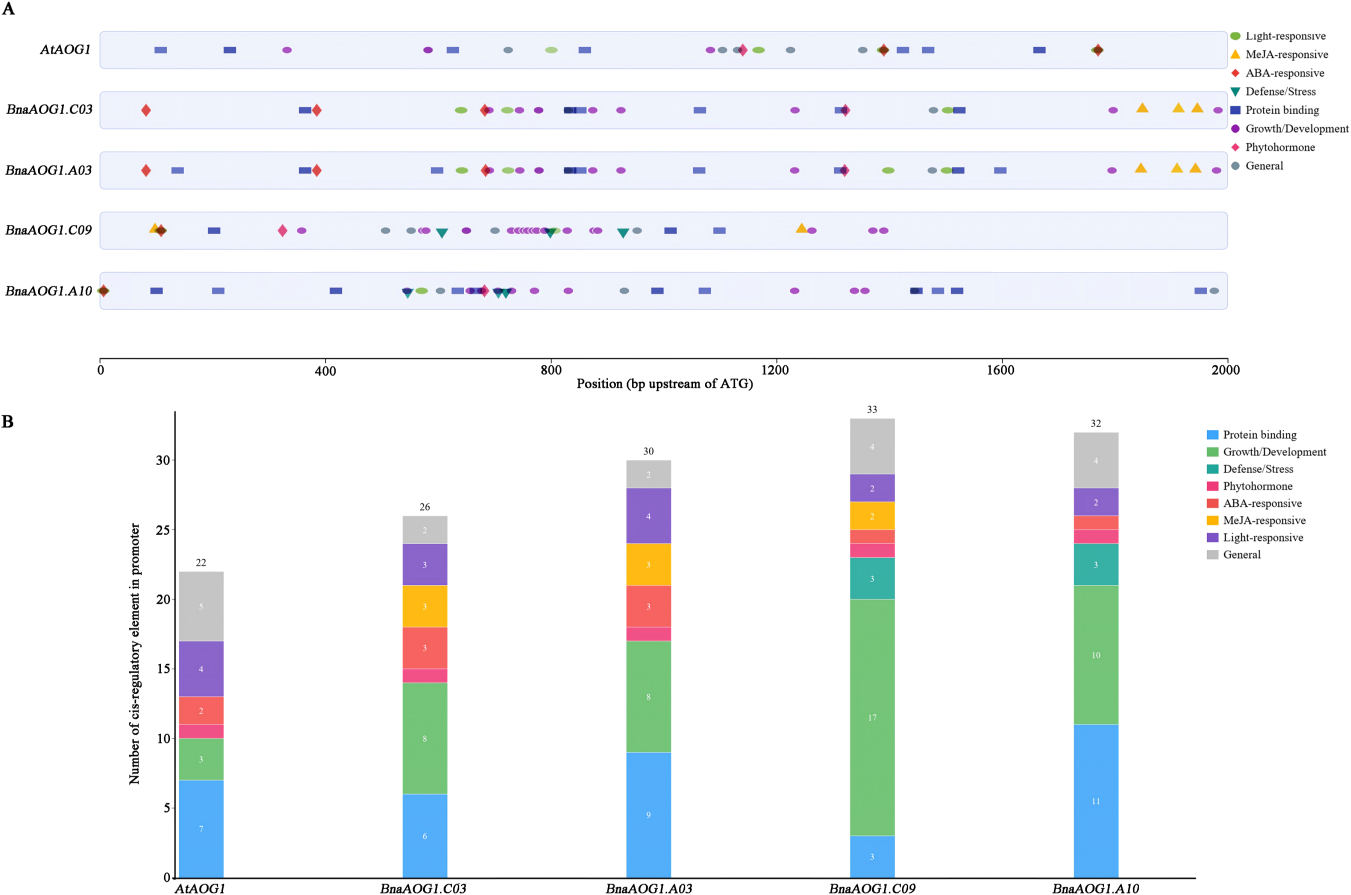
*Cis-regulatory* elements in the promoters of *BnaAOG1s.* **(A)**, distribution of *cis-regulatory* elements in the possible promotor regions of *BnaAOG1s*. **(B)**, number of different kinds of *cis-regulatory* elements.

To further understand the potential transcriptional regulation of *BnaAOG1s*, the *cis-regulatory* elements present in the region 2.0 kb upstream of *BnaAOG1s* were predicted. According to their biological functions, the *cis-regulatory* elements identified could be divided into six categories: Light-responsive elements, phytohormone responsive elements, defense and stress responsive elements, growth and development, general elements (AT-rich element) and protein binding sites (**Figure 3A**). Among them, growth and development, and light-responsive elements account for the largest proportion in the promoter regions of *BnaAOG1s* (**Figure 3B**). In addition, elements responsive to various phytohormones, including methyl jasmonate (MeJA), abscisic acid (ABA), and other phytohormone responsive elements were identified. In *BnaAOG1.A10* and *BnaAOG1.C09*, defense and stress responsive elements were predicted to exist in the promoter regions.

### Expression profiles of *BnaAOG1s*

**Figure 4:**
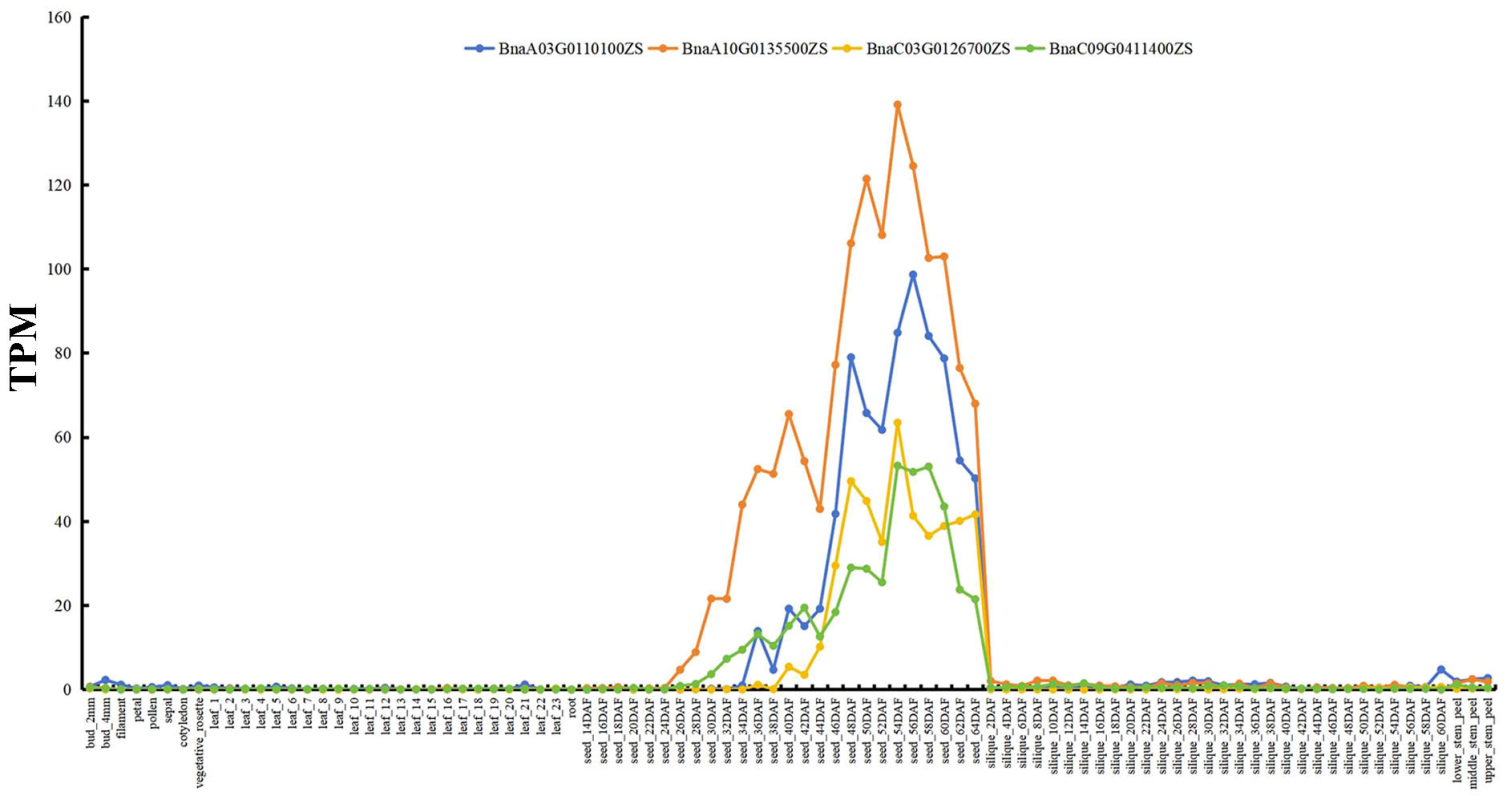
Expression of *BnaAOG1s* in various tissues of *B. napus* (ZS11). Gene expression level: TPM, Transcripts Per Million.

Transcriptome data from ZS11 showed that expression of *BnaAOG1s* was minimal in most tissues, with the notable exception of developing seeds, where high transcript abundance was observed (**Figure 4**; **Supplementary Table 2**). In contrast, *AtAOG1* in Arabidopsis was expressed ubiquitously in different tissues including roots, stems, leaves, seedlings, inflorescences and siliques, but much higher in reproductive organs (inflorescences and siliques), with GUS signals strongest in pollen grains and ovules (Cui et al., 2015). Thus, the expression pattern of *BnaAOG1s* in rapeseed only partially recapitulates that of *AtAOG1*, and clear expression divergence has occurred among the homoeologous copies.

### Haplotype analysis of *BnaAOG1s*

To further predict the roles of different *BnaAOG1s* in regulating TSW and SL, we used the online tool of the BnIR website to analyze the correlation between natural variations in *BnaAOG1s* and the TSW and SL phenotypes in a natural population.

**Figure 5:**
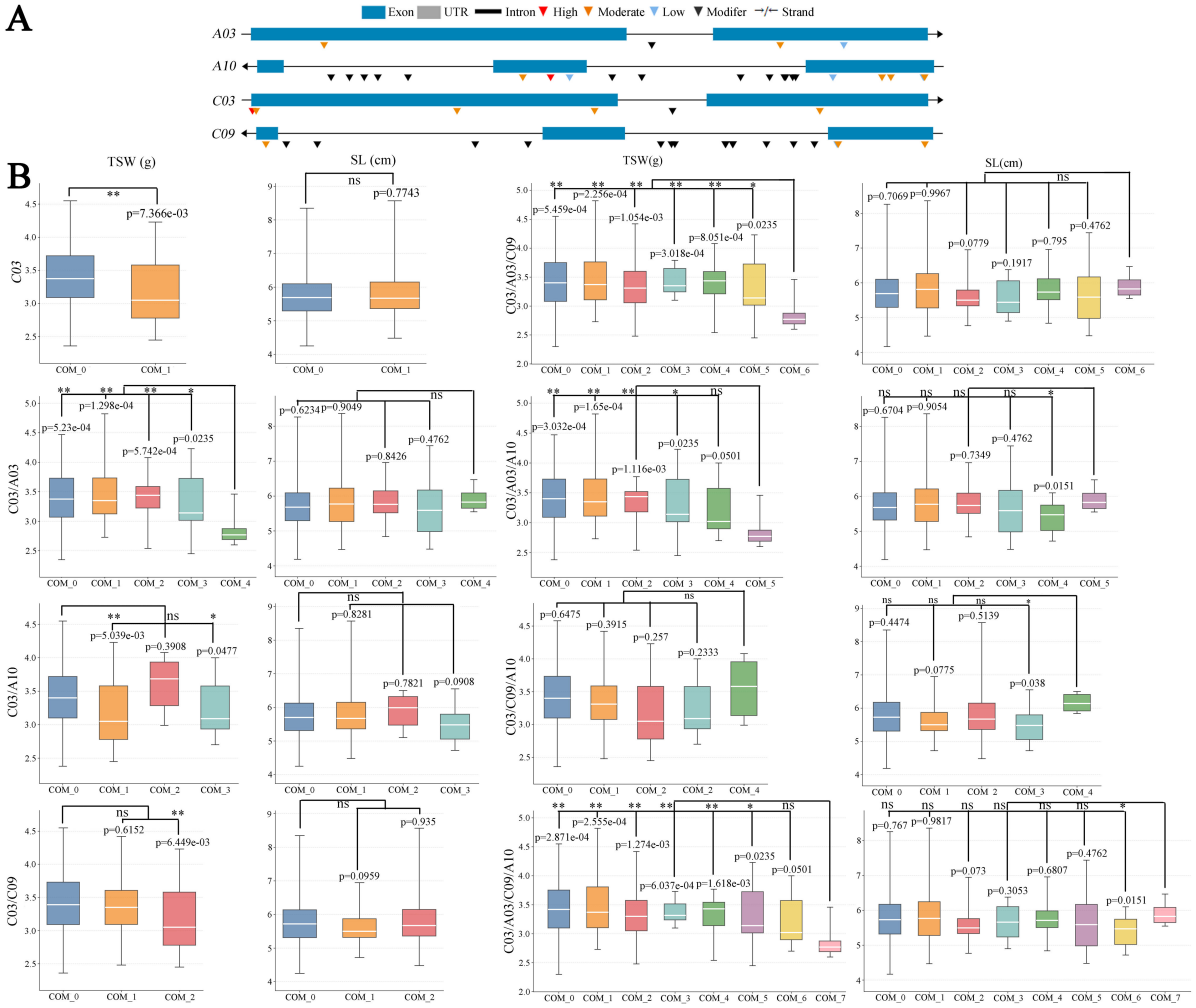
Haplotype analysis of *BnaAOG1s.* **(A)**, Summary of the haplotypes in *BnaAOG1s*. The diagram shows the *BnaAOG1*s structure and the positions of the four polymorphisms identified. **(B)**, Thousand seed weight and silique length across *B. napus* accessions with different haplotype combinations in *BnaAOG1s*. Asterisks indicate significant differences, as determined by One-Way ANOVA test; *P < 0.05, **P < 0.01.

We identified 4, 6, 21, and 18 polymorphic variations in *BnaAOG1.A03*, *BnaAOG1.C03*, *BnaAOG1.A10*, and *BnaAOG1.C09*, respectively. Among these, 2, 5, 5, and 3 were missense or frameshift polymorphisms (**Figure 5A**; **Supplementary Table 5**). Phenotypic analysis of TSW revealed that, except for *BnaAOG1.C03*, none of the single-gene variations caused significant changes in TSW. However, when haplotype variations of different genes were considered together, significant differences in TSW emerged among different variation types, and different gene combinations exhibited distinct effects on TSW (**Figure 5B**; **Supplementary Figure 2**). Notably, the combination of *BnaAOG1.C03* and *BnaAOG1.A03* led to a more pronounced change in TSW compared to the single-gene variation of *BnaAOG1.C03*, and this enhanced effect was also observed in combinations involving more genes, whereas combining *BnaAOG1.C03* with each of the other two genes individually did not produce such an effect. Regarding silique length, no single-gene haplotype variation caused significant changes. However, two-gene haplotype combinations of *BnaAOG1.A10* with *BnaAOG1.A03* or *BnaAOG1.C09* resulted in significant changes in SL, with the combination involving *BnaAOG1.C09* showing the most significant effect. These results indicate that *BnaAOG1.C03* is a major gene regulating TSW, and there is a synergistic effect between *BnaAOG1.A03* and *BnaAOG1.C03*. In contrast, silique length appears to be influenced by more genes or exhibits stronger environmental interactions, with *BnaAOG1.A10* and *BnaAOG1.C09* showing a clear trend effect in regulating SL.

### CRISPR/Cas9 editing of *BnaAOG1.A03*/*C03* and generation of mutants

**Figure 6:**
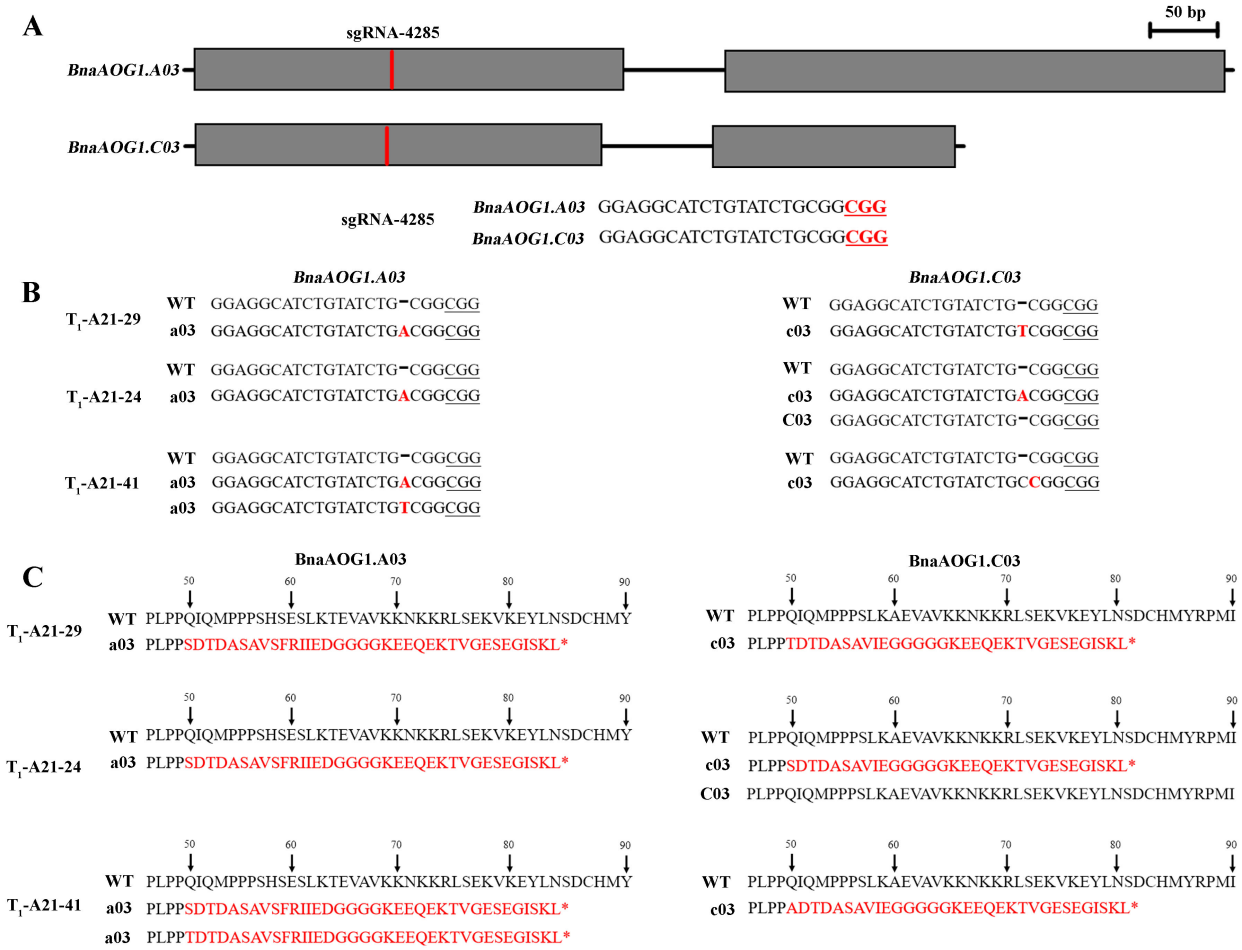
CRISPR-Cas9-induced *BnaAOG1s* gene editing in *B. napus*. **(A)**, Schematic representation of *BnaAOG1s* gene structure and target site. Exons and introns of *BnaAOG1s* are indicated with gray rectangles and black lines, respectively. The nucleotides of target site are shown in capital letters and the protospacer adjacent motif (PAM) site is underlined. **(B)**, Nucleotide sequences at the target site in the T_1_ mutant lines. The mutated alleles are shown below the WT sequence. The nucleotides of target site are indicated with black capital letters. The PAM site is underlined. The red dashes indicate deleted nucleotides. The red capital letters indicate inserted or substituted nucleotides. **(C)**, Amino acid sequence at the target site in the T_1_ mutant lines. Red star indicated premature termination of translation. The red capital letters indicate frameshift mutation. S represents sgRNA; WT represents wild-type; a03 represents *BnaAOG1.A03*; c03 represents *BnaAOG1.C03*.

To investigate the function of *BnaAOG1s* in silique and seed development, we targeted *BnaAOG1s* using CRISPR/Cas9 (**Figure 6A**). To date, we have successfully obtained homozygous editing lines only for *BnaAOG1.A03* and *BnaAOG1.C03* (**Figure 6**).

A total of 35 T_0_ plants were regenerated, and 26 were confirmed as positive by PCR (positive rate 74.3%). High−throughput Hi−TOM sequencing of the target site revealed that 9 of the 26 positive plants (34.6%) carried editing events. The editing types were predominantly single−base insertions (mostly adenosine) (**Figure 6B**).

T_1_ seeds were obtained by self−pollination of T_0_ edited plants and were grown under speed−breeding conditions for generation advancement (Song et al., 2022). Genotyping by Hi−TOM using specific primers identified homozygous edited lines. In the A21 family, two homozygous double−mutant lines were obtained: T_1_−A21−29 and T_1_−A21−41. Sanger sequencing confirmed that in T_1_−A21−29, both *BnaAOG1.A03* and *BnaAOG1.C03* carried a single−base (A/C) insertion in the first exon (**Figure 6B**), causing frameshifts and premature translation termination (**Figure 6C**). In T_1_−A21−41, *BnaAOG1.A03* had two different single−base insertion events (heterozygous) and *BnaAOG1.C03* had a homozygous single−base (C) insertion.

### Phenotypic analysis of homozygous mutants

To evaluate the effect of the double mutation in *BnaAOG1.A03* and *BnaAOG1.C03* on silique and seed development, T_1_−A21−24, T_1_−A21−29, and T_1_−A21−41 mutants and wild−type Xiaoyun were grown together in the greenhouse, and key agronomic traits were measured at maturity (**Supplementary Figure 3**).

**Figure 7:**
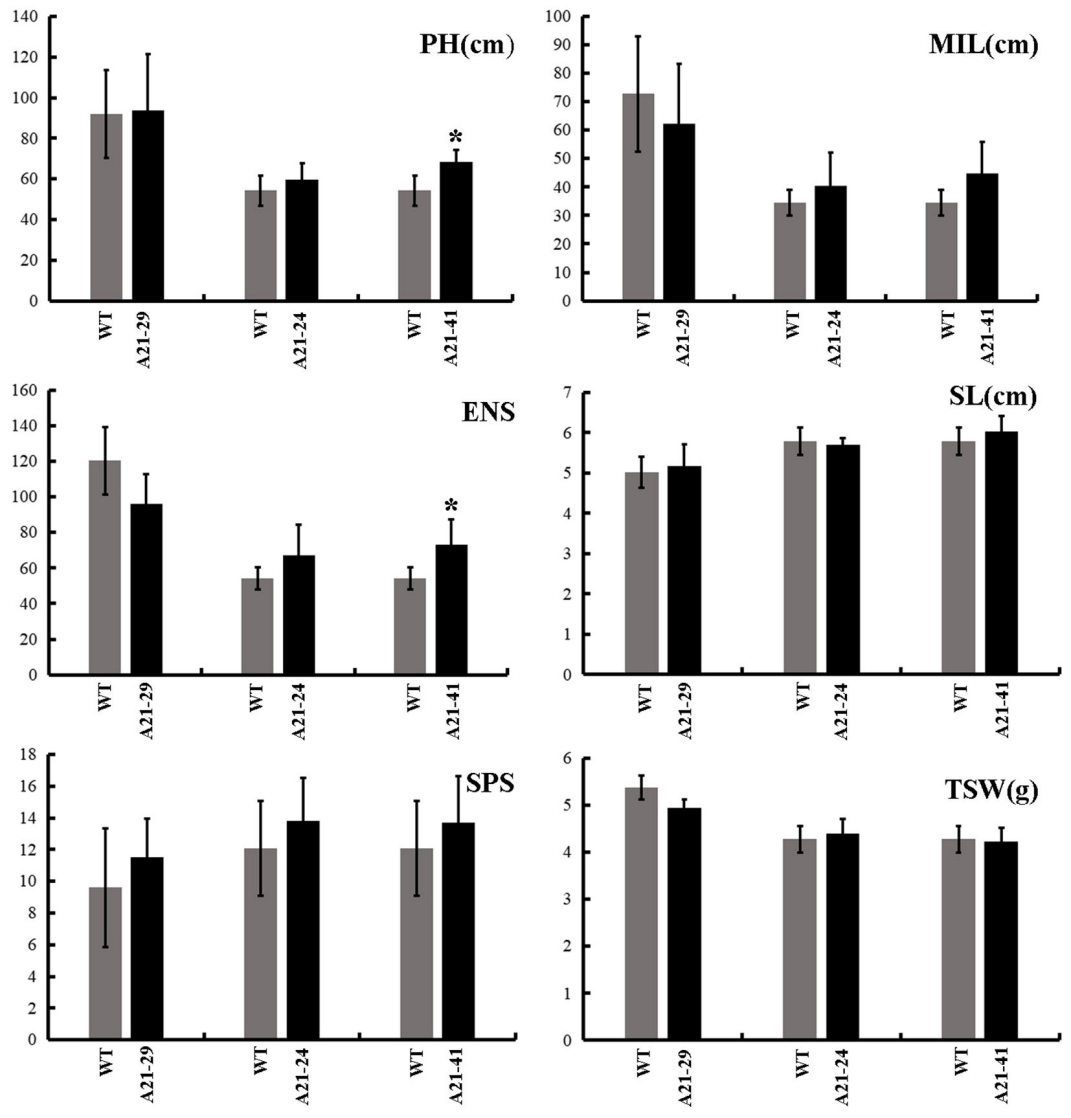
Investigation on important agronomic traits of T_1_ homozygous mutant lines for *BnaAOG1.A03* and *BnaAOG1.C03*. Genotype for A21-24: aacC; Genotype for A21-29: aacc; Genotype for A21-41: aacc; PH, plant height; MIL, length of main inflorescence; ENS, effective silique number per plant; SL, silique length; SPS, seeds number per silique; TSW, thousand seed weight. *p<0.05.

No significant differences were observed between the mutant and wild type with respect to silique length, SPS, or TSW (**Figure 7**). Plant height and main inflorescence length were also similar to wild type. Although the effective silique number per plant showed a slight difference, it was not statistically significant. Flowering time and growth duration were not altered in the mutant.

To rule out possible effects of genetic background or editing type, we also analyzed two additional lines: T_1_−A21−24 (*BnaAOG1.A03* homozygous, *BnaAOG1.C03* heterozygous) and T_1_−A21−41 (*BnaAOG1.A03* heterozygous, *BnaAOG1.C03* homozygous). All these lines showed no significant differences in silique length, SPS, or TSW compared to the wild type (**Figure 7**). However, T_1_−A21−41 exhibited significantly increased plant height and effective silique number per plant.

## Discussion

### Functional divergence and redundancy of *AOG1s*

In Arabidopsis, *AOG1* is essential for gametophyte development (Cui et al., 2015). The *aog1* mutation arrests male gametophytes at the uninucleate microspore stage (≈60% degenerated pollen) and female gametophytes at the uninucleate embryo sac stage, leading to severely shortened siliques (≈50% reduction) and a sharp decline in seed set (≈8.77% vs. 99.24% in wild type). AOG1 is a nuclear protein of unknown biochemical function, possibly affecting gametophytic mitosis by regulating cell−cycle genes such as *DUO1* and *CDKA;1*. Its strong expression in pollen grains and ovules suggests a direct role in these reproductive cells (Cui et al., 2015).

In Arabidopsis, *AOG1* exists as a single−copy gene, such that its loss−of−function yields a complete phenotype. In contrast, *B. napus* contains four homoeologous copies, making functional analysis more complex. Expression analysis demonstrated that *BnaAOG1s* differ from *AtAOG1*, being predominantly highly expressed in seeds while exhibiting very low expression levels in other tissues (**Figure 4**; Cui et al., 2015). The *cis-regulatory* element analysis revealed distinct regulatory architectures between *BnaAOG1s* and *AtAOG1* that reflect functional divergence following polyploidization (**Figure 3**). Notably, all four *BnaAOG1* genes exhibited a substantial expansion in the total number of *cis-regulatory* elements compared to *AtAOG1* (22 elements), ranging from 26 in *BnaAOG1.C03* to 33 in *BnaAOG1.C09*, representing an increase of 18-50%. This observation is consistent with the notion that polyploidization events are often accompanied by increased regulatory complexity, which may contribute to phenotypic diversification and adaptive evolution (Chalhoub et al., 2014; Song et al., 2020). Further analysis revealed significant differences in the composition of cis-elements among the four *BnaAOG1* genes. Most notably, *BnaAOG1.A10/C09* contains significantly more growth and development-related elements than *BnaAOG1.A03/C03*, and only *BnaAOG1.A10/C09* possesses three stress-response elements, suggesting its potential role in coordinating seed or silique development with stress adaptation mechanisms (**Figure 3**). Sequence alignment and phylogenetic tree analysis revealed possible functional divergence between *BnaAOG1.A03/C03* and *BnaAOG1.A10/C09* (**Figures 1**, **2**). Haplotype analysis of natural populations further confirmed that *BnaAOG1.A03/C03* tend to regulate thousand-seed weight, whereas *BnaAOG1.A10/C09* exhibit a stronger trend effect in regulating silique length (**Figure 5**; **Supplementary Figure 2**). These results suggest that the function of *BnaAOG1s* may differ somewhat from that of *AtAOG1*, and that *BnaAOG1.A03/C03* and *BnaAOG1.A10/C09* may have undergone subfunctionalization.

Subsequently, we generated homozygous mutant lines for *BnaAOG1.A03* and *BnaAOG1.C03* via CRISPR/Cas9−mediated gene editing, and their plant architecture, silique, and seed traits were systematically evaluated. In stark contrast to the severe gametophytic defects, shortened siliques, and markedly reduced seed set reported for the *aog1* mutant in Arabidopsis (Cui et al., 2015), the rapeseed mutants exhibited no overt abnormalities in SL, SPS, or TSW. Given that the other two copies (A10 and C09) were not successfully edited, it remains possible that these remaining copies provide sufficient AOG1 function to sustain normal gametophyte development and silique growth. Thus, the possibility of functional redundancy among different *BnaAOG1* copies cannot yet be ruled out. Further construction of diverse mutant lines of *BnaAOG1s* and phenotypic analysis under field conditions is required for comprehensive systematic analysis to investigate functional differentiation and redundancy between these copies.

Surprisingly, among the three homozygous double-mutant lines generated in this study, T_1_-A21–41 exhibited a notably distinct phenotype characterized by significantly increased PH and ENS compared with WT, whereas T_1_-A21–29 and T_1_-A21–24 showed no significant alterations in these architectural traits relative to their respective wild-type controls (**Figure 7**). Sequence analysis revealed that the mutation configuration in T_1_-A21–41 differed markedly from the other two lines. *BnaAOG1.A03* carried two distinct single-base insertion alleles (heteroallelic), indicating differential editing events on the two homoeologous chromosomes, while *BnaAOG1.C03* harboured a homozygous single-base insertion. In contrast, T_1_-A21–29 harboured identical single-base insertions in both copies of both target genes, and T_1_-A21–24 was heterozygous at *BnaAOG1.C03*. Importantly, the remaining agronomic traits-MIL, SL, SPS, and TSW-showed no statistically significant differences between any of the mutant lines and their corresponding wild-type controls across all three lineages (**Figure 7**). In polyploid genomes, homoeologous gene copies often exhibit functional redundancy through compensatory pathways, whereby the remaining unedited copies sustain sufficient gene function to maintain wild-type phenotypes (Adams and Wendel, 2005; Lynch and Conery, 2000; Flagel and Wendel, 2009). We hypothesize that the heteroallelic configuration in A21–41 may impose a more severe disruption of residual *BnaAOG1.A03* copy function than homozygous identical mutations, potentially destabilizing compensatory mechanisms that normally operate under incomplete gene inactivation in allopolyploid crops (Hu et al., 2026). Alternatively, asymmetric expression of heteroallelic transcripts could disrupt regulatory networks governing plant architecture, a phenomenon documented in other CRISPR-edited multi-copy gene systems in *B. napus* (Yang et al., 2018). However, given that the principal yield-related traits-SL, SPS, and TSW-remained unchanged across all mutant lines including A21-41, the increase in PH and ENS observed specifically in this line are unlikely to be directly attributable to the disruption of *BnaAOG1.A03* and *BnaAOG1.C03* per se, but may rather reflect residual genetic background variation segregating within the edited population. Of course, the current results cannot rule out the potential influence of unknown T-DNA insertions, nor can they fully exclude whether differences in the microenvironment of comprehensive speed breeding platforms account for these growth variations. In the future, we will continue gene editing and screening/purification of *BnAOG1s* to obtain additional purified lines with diverse editing profiles and free of T-DNA insertions, followed by phenotypic analysis under field conditions to acquire more reliable data and better elucidate the functional interactions among different *BnAOG1s* copies.

### Implications for functional gene studies in polyploid crops

This study provides a typical case of homoeologous gene functional analysis in a polyploid crop. A gene with essential functions in the model plant Arabidopsis may show reduced or shifted functions in the polyploid crop *B. napus* due to gene redundancy or functional divergence. This phenomenon is widespread in polyploid genomes, such as in *BnaMAX1*, lines harboring only a single functional allele loss (S1-5) displayed a completely wild-type architecture, whereas lines with all four BnaMAX1 alleles simultaneously knocked out (S1–8 and S1-11) exhibited dramatic semi-dwarf phenotypes (plant height reduced by 31.9%-36.5%), increased branching (from 3 to 9 primary branches), and a 29.9%-31.3% increase in seed yield per plant (Zheng et al., 2020). Therefore, functional genomic studies in polyploid crops cannot rely solely on knowledge from model plants; direct gene editing and multi−copy mutant analysis are necessary. The CRISPR/Cas9 system used here offers an efficient tool for dissecting the functions of multi−copy genes (Lu et al., 2017; Bai et al., 2020). Moreover, integration of comprehensive speed breeding platforms can significantly shorten generation times and accelerate the acquisition of multi−copy mutants and phenotypic evaluation (Song et al., 2022).

Our findings also suggest that candidate genes for molecular breeding in rapeseed should be selected with caution. For functionally conserved genes (e.g., *UBP15*, *EOD1*), favorable alleles can be directly used for genetic improvement (Gu et al., 2023a; Gu et al., 2023b). For genes that may have undergone functional divergence (e.g., *AOG1*), more extensive functional validation is required.

## Conclusion

In summary, through bioinformatics analysis, we demonstrated that *BnaAOG1s* may function differently from *AtAOG1*, and that *BnaAOG1.A03/C03* and *BnaAOG1.A10/C09* may have undergone further subfunctionalization. However, this conclusion is limited by the insufficient number of mutant lines available. The present experimental results can only indicate that simple genetic modifications for *BnaAOG1.A03* and *BnaAOG1.C03* do not significantly alter the development of rapeseed seeds or siliques. The four *BnaAOG1s* copies may govern the development of rapeseed seeds and siliques through interaction mechanisms that are far more complex than anticipated. This study provides preliminary evidence for elucidating the functional role of *BnaAOG1s* in regulating the development of rapeseed seeds and siliques. Future efforts to generate more types of mutants without T-DNA insertions and conduct multi−environment phenotypic analyses will help fully elucidate the biological functions of *BnaAOG1s* in rapeseed.

## Data Availability

The datasets presented in this study can be found in online repositories. The names of the repository/repositories and accession number(s) can be found in the article/**Supplementary Material**.

## Supplementary material

Supplementary Figure 1 Analysis of protein sequence identity between different copies of BnaAOG1s.

Supplementary Figure 2 Analysis of protein sequence identity between different copies of *BnaAOG1s*.

Supplementary Figure 3 Representative photographs for different stage of T_1_-A21-24, T_1_-A21-26, T_1_-A21–41 and WT. A. Seelings of T_1_-A21-24, T_1_-A21-26, T_1_-A21–41 and WT; B, Silique of T_1_-A21-24, T_1_-A21-26, T_1_-A21–41 and WT; C. Mature plant of T_1_-A21-24, T_1_-A21-26, T_1_-A21–41 and WT; D. seed of T_1_-A21-24, T_1_-A21-26, T_1_-A21–41 and WT.
