# Supplementary figures and images for: CRISPR/Cas9-mediated mutagenesis of BnaAOG1s reveals functional divergence in silique and seed development in Brassica napus L
Source: Front Plant Sci. 2026 Jul 20;17:1862587. doi: 10.3389/fpls.2026.1862587 (PMC13429740; doi:10.3389/fpls.2026.1862587)

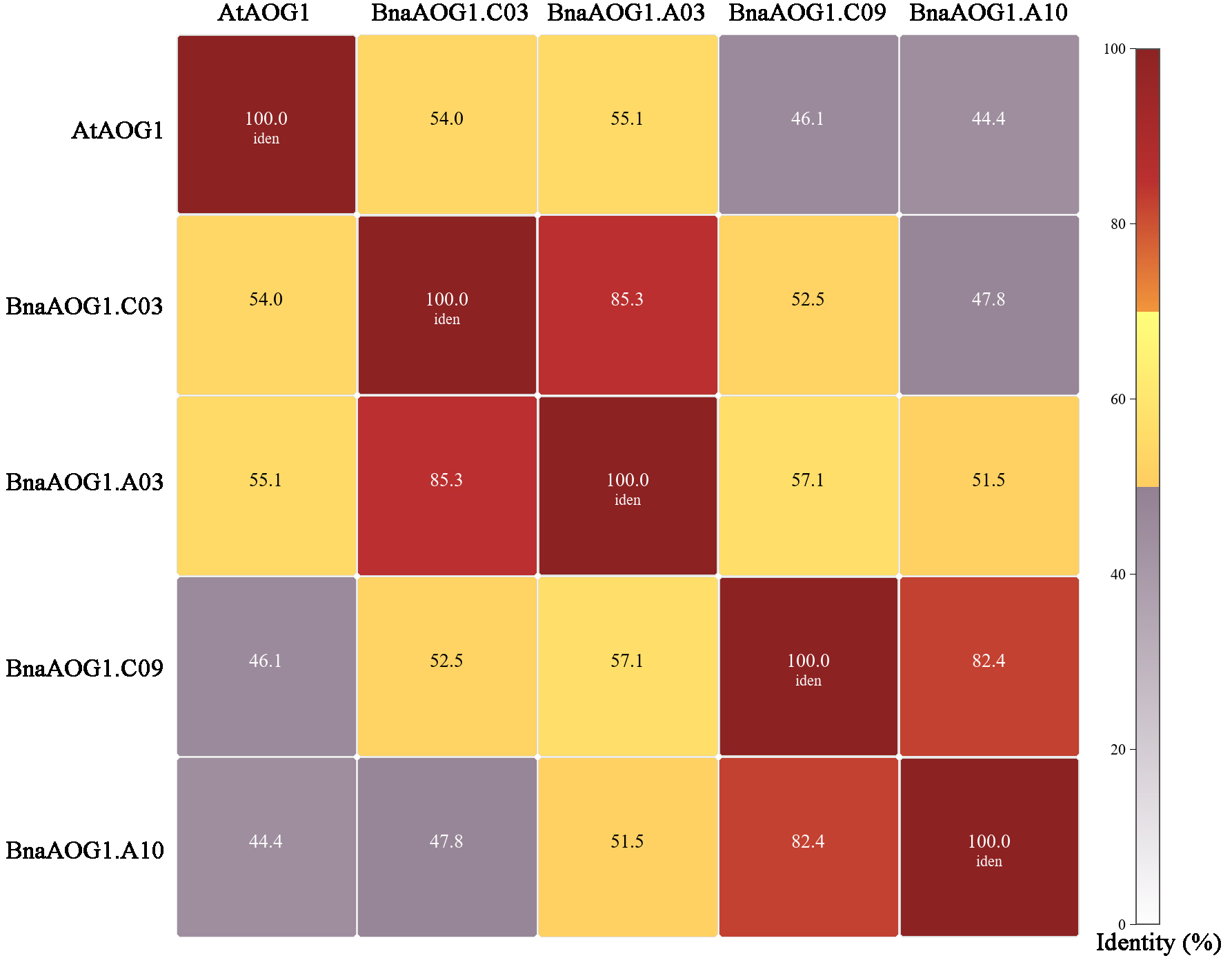

Supplement: Supplementary Figure 1 — Analysis of protein sequence identity between different copies of BnaAOG1s. [file Image1.jpeg]

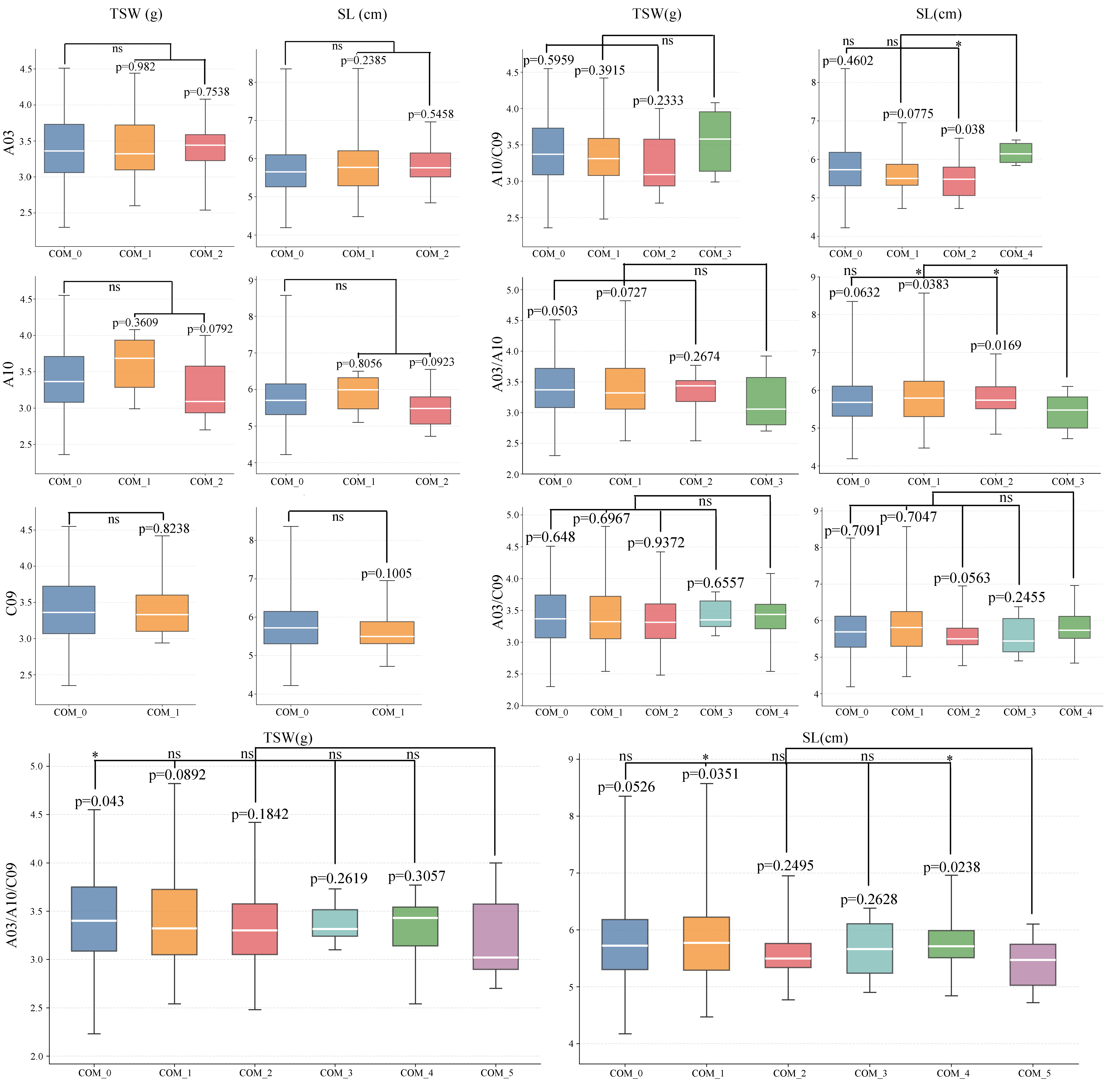

Supplement: Supplementary Figure 2 — Analysis of protein sequence identity between different copies of BnaAOG1s. [file Image2.jpeg]

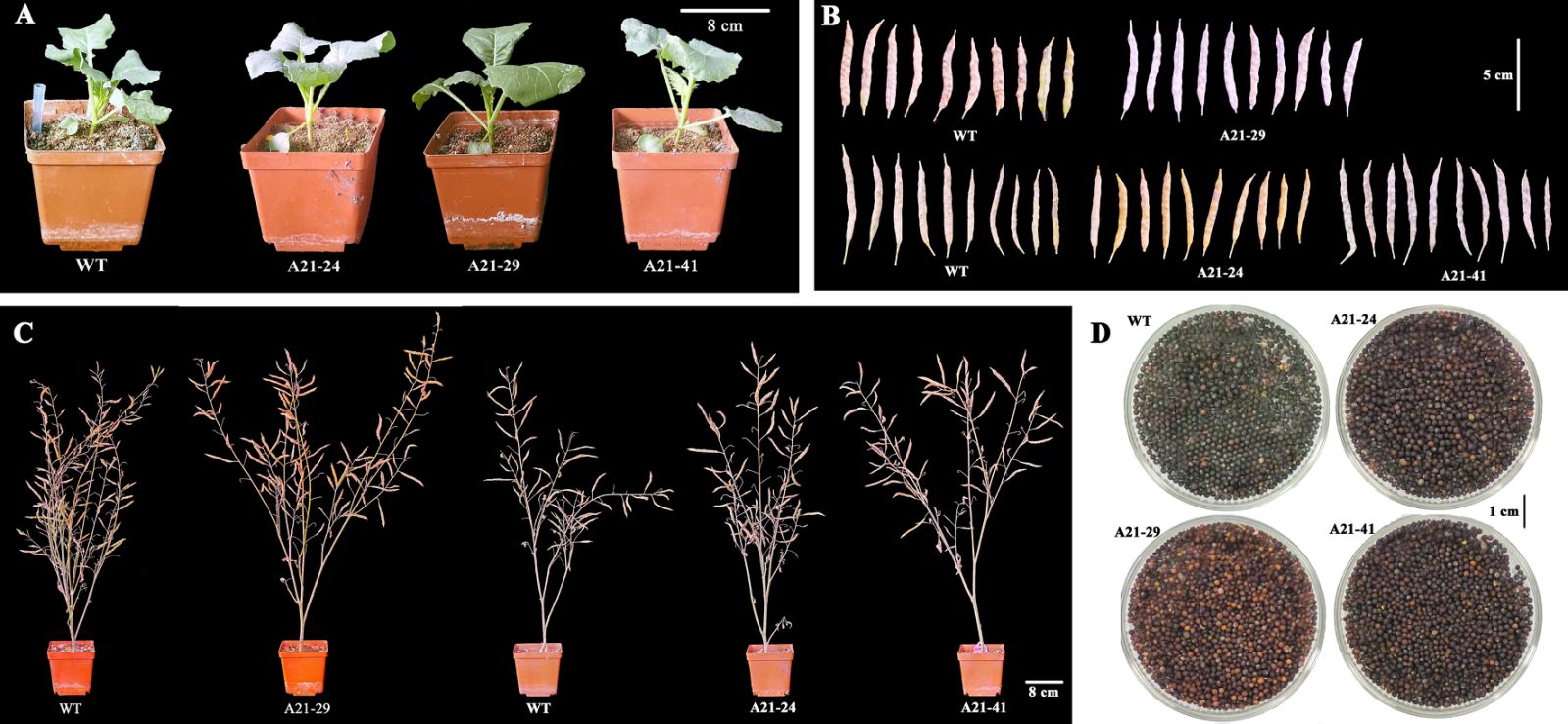

Supplement: Supplementary Figure 3 — Representative photographs for different stage of T1-A21-24, T1-A21-26, T1-A21–41 and WT. A. Seelings of T1-A21-24, T1-A21-26, T1-A21–41 and WT; B, Silique of T1-A21-24, T1-A21-26, T1-A21–41 and WT; C. Mature plant of T1-A21-24, T1-A21-26, T1-A21–41 and WT; D. seed of T1-A21-24, T1-A21-26, T1-A21–41 and WT. [file Image3.jpeg]
